# Effects of Dietary Glutamine on the Homeostasis of CD4+ T Cells in Mice with Dextran Sulfate Sodium-Induced Acute Colitis

**DOI:** 10.1371/journal.pone.0084410

**Published:** 2014-01-09

**Authors:** Yuan-Chin Hsiung, Jun-Jen Liu, Yu-Chen Hou, Chiu-Li Yeh, Sung-Ling Yeh

**Affiliations:** 1 School of Nutrition and Health Sciences, Taipei Medical University, Taipei, Taiwan; 2 School of Medical Laboratory Science and Biotechnology, Taipei Medical University, Taipei, Taiwan; 3 Department of Surgery, National Taiwan University Hospital, Taipei, Taiwan; 4 Department of Food and Nutrition, Chinese Culture University, Taipei, Taiwan; Cincinnati Children's Hospital Medical Center, University of Cincinnati College of Medicine, United States of America

## Abstract

This study investigated the effects of dietary glutamine (Gln) on T-helper (Th) and T regulatory (Treg) cell homeostasis and colonic inflammatory mediator expression in mice with dextran sulfate sodium (DSS)-induced colitis. Mice were randomly assigned to 4 groups with 2 normal control (C and G) and 2 DSS-treated groups (DC and DG). The C and DC groups were fed a common semipurified diet, while the G and DG groups received an identical diet except that part of the casein was replaced by Gln, which provided 25% of the total amino acid nitrogen. Mice were fed the diets for 10 days. On day 6, mice in the normal control groups were given distilled water, while those in the DSS groups were given distilled water containing 1.5% DSS for 5 d. At the end of the experiment, the mice were sacrificed for further examination. Results showed that DC group had higher plasma haptoglobin, colonic weight, immunoglobulin G, inflammatory cytokine and nuclear factor (NF)-κB protein levels. Gln administration lowered inflammatory mediators and NF-κB/IκBα ratio in colitis. Compared with the DC group, the percentages of interleukin-17F and interferon-γ in blood and transcription factors, T-bet and RAR-related orphan receptor-γt, gene expressions in mesenteric lymph nodes were lower, whereas blood Foxp3 was higher in the DG group. Also, DG group had lower colon injury score. These results suggest that Gln administration suppressed Th1/Th17 and Th-associated cytokine expressions and upregulated the expression of Tregs, which may modulate the balance of Th/Treg and reduce inflammatory reactions in DSS-induced colitis.

## Introduction

Inflammatory bowel disease (IBD), which includes Crohn's disease (CD) and ulcerative colitis (UC), refers to chronic inflammatory disorders that may affect the entire gastrointestinal tract [1]. The precise etiology of IBD remains unidentified [2]. A recent study indicated that genetic factors, epithelial barrier functions, gut microbiota, and the host immune system are involved in the development and course of IBD [3].

CD4^+^ helper T (Th) cells play major roles in both the induction and persistence of IBD by producing proinflammatory cytokines. Th cells are traditionally divided into Th1 and Th2 subsets, characterized by distinct cytokines and effector functions. Interferon (IFN)-γ is produced by Th1 lymphocytes, and interleukin (IL)-4 is a Th2 cytokine. Classically, CD is considered a Th1-mediated disease, whereas UC exerts a Th2-like response [4]–[6]. A recent study suggested that the Th17-cell-driven immune response, which produces excess IL-17A, IL-17F, and IL-22, plays a critical role in the pathogenesis of both CD and UC [7]. An additional Th cell subset which produces IL-22 was identified and designated Th22. Th22 may also participate in the immune response of IBD, but the exact role remains ambiguous [8], [9]. Regulatory T (Treg) cells are a distinct T cell subset with opposing actions to Th17. Treg cells are implicated in suppressing an excessive T cell response [10]. The intestinal mucosa is normally maintained in an equilibrium state in which the protective immunity and tolerance to self-antigens and commensal bacteria are balanced. This tolerance is maintained by Treg cells in the gut by inhibiting the proliferation and effector functions of other T cells [11]. Recent studies indicated that IBD is associated with a decrease in Treg cells and increase in Th17 cells in peripheral blood [12], [13].

Glutamine (Gln) is the most abundant free amino acid in the plasma and tissue pool. It serves as an important fuel source for rapidly dividing cells, especially lymphocytes and enterocytes. Although it is a non-essential amino acid, many studies showed that Gln has immunomodulatory properties and is considered conditionally essential for patients with catabolic conditions [14]–[17]. Shiomi et al. [18] reported that Gln levels of serum and colon tissues were significantly lower in the acute phase of colonic inflammation, and Gln supplementation attenuated the degree of microscopic injury induced by dextran sulfate sodium (DSS). Previous studies showed that Gln therapy improves outcomes of in vitro and in vivo experimental colitis models, and is able to attenuate proinflammatory mediator expressions in experimental colitis [19], [20]. A recent study performed by our laboratory found that pretreatment with alanyl-Gln injection suppresses Th cell-associated cytokine expression and reduces inflammatory responses in mice with acute DSS-induced colitis [21]. However, studies concerning the effect of dietary Gln on the balance of Th/Treg cells and colonic tissue damage in IBD are rare. Therefore, this study investigated the effects of dietary Gln supplementation on Th/Treg cell homeostasis, colonic cell apoptosis and inflammatory mediator expression in mice with DSS-induced acute colitis.

## Materials and Methods

### Animal preparations

Male C57BL/6 mice at 8∼12 weeks old and weighing 22∼25 g at the beginning of the experiment were used in this study. All mice were housed in cages in a temperature- and humidity-controlled room and were allowed free access to a standard chow diet for 1 wk before the study. Care of laboratory animals was in full compliance with the *Guide for the Care and Use of Laboratory Animals* (National Research Council, 1996), and protocols were approved by the institutional Animal Care and Use Committee of Taipei Medical University.

### Experimental procedures

Thirty-two mice were assigned to 4 groups with 2 normal control (C and G) and 2 DSS-treated groups (DC and DG). The C and DC groups were fed a common semipurified diet, while the G and DG groups received an identical diet except that part of the casein was replaced by Gln, which provided 25% of total amino acid nitrogen. The 2 diets were isocaloric and isonitrogenous (Table 1). The amount of GLN used is known to have an immunomodulatory effect on catabolic conditions [22]. The mice were not paired fed because the food intakes between the 2 control groups and 2 DSS-treated groups were comparable. The diets were fed for 10 days. On day 6, mice in the normal control groups were given distilled water, and those in the DSS groups were given distilled water containing 1.5% (wt/vol) DSS (MW 40 kDa; MP Biomedicals, Solon, OH) for 5 d. During the experimental period, the body weight (BW) was recorded daily. At the end of the experiment (day 11), mice were anesthetized with an intraperitoneal Zoletil (20 mg/kg) injection and sacrificed by cardiac puncture. Whole blood was centrifuged at 3000×*g* for 10 min at 4°C to obtain plasma. The mesenteric lymph nodes (MLNs) were removed and processed for RNA isolation. The colon was cut close to the ileocecal valve, and the length and weight were measured. The luminal content of the colon was collected by flushing each specimen with 2 ml of ice-cold phosphate-buffered saline (PBS). Colon lavage fluid (CLF) was centrifuged at 2000×*g* for 10 min at 4°C, and supernatants were collected and kept at −80°C until being processed for further analyses. Sections (1 cm) of the distal colon were cut out and fixed with buffered 4% paraformaldehyde for a histopathological examination.

**Table 1:** Composition of the experimental diets (g/kg).

Component	Control diet	Glutamine diet
Soybean oil	100	100
Casein	200	150
Glutamine	0	41.7
Salt mixture†	35	35
Vitamin mixture‡	10	10
Methyl cellulose	31	31
Choline bitartrate	2.5	2.5
Methionine	3	3
Corn starch	626.8	618.5

The salt mixture contained the following (mg/g): calcium phosphate dibasic, 500; sodium chloride, 74; potassium sulfate, 52; potassium citrate monohydrate, 20; magnesium oxide, 24; manganese carbonate, 3.5; ferric citrate, 6; zinc carbonate, 1.6; curpric carbonate, 0.3; potassium iodate, 0.01; sodium selenite, 0.01; and chromium potassium sulfate, 0.55.

The vitamin mixture contained the following (mg/g): thiamin hydrochloride, 0.6; riboflavin, 0.6; pyridoxine hydrochloride, 0.7; nicotinic acid, 3; calcium pantothenate, 1.6; D-biotin, 0.05; cyanocobalamin, 0.001; retinyl palmitate, 1.6; DL-α-tocopherol acetate, 20; cholecalciferol, 0.25; and menaquinone, 0.005.

### Measurements of haptoglobin in plasma

Plasma samples were used to determine the concentration of haptoglobin by an enzyme-linked immunosorbent assay (ELISA) kit (ICL, Newberg, OR). Procedures followed the manufacturer's instructions.

### Distribution of T lymphocyte subpopulations in blood

To determine the phenotypes of helper T lymphocytes in the blood, 100-µl aliquots of whole blood were incubated with Pacific blue-conjugated anti-mouse CD4 (BD Biosciences, San Jose, CA). Another 100 µl of whole blood was incubated with Pacific blue-conjugated anti-mouse CD4 and APC-conjugated anti-mouse CD25 (eBioscience, San Diego, CA) for 30 min to analyze the Treg cell percentage. After lysing red blood cells, the remaining leukocytes were fixed and permeated for intracellular cytokine staining. The following antibodies (Abs) were used for intracellular cytokine staining: Alexa Fluor® 488-conjugated anti-mouse IL-4 (eBioscience), Alexa Fluor® 647-conjugated anti-mouse IFN-γ (Biolegend, San Diego, CA), fluorescein isothiocyanate (FITC)-conjugated anti-mouse IL-17A (eBioscience), phycoerythrin (PE)-conjugated anti-mouse IL-17F (eBioscience), and PerCP-eFluor® 710-conjugated anti-mouse IL-22 (eBioscience). For intracellular staining of forhead box p3 (Foxp3), leukocytes were fixed and permeated with Foxp3 staining buffer (eBioscience) and PE-conjugated anti-mouse Foxp3 (Biolegend). Cells were analyzed with a FACS *Canto II* flow cytometer (BD Biosciences). Lymphocytes were gated with characteristic low forward- and side-scatter profiles. Phenotypes of Th cells are presented as percentages of Th-associated cytokine-expressing cells among Th cells (CD4^+^ lymphocytes). Treg cells are presented as a percentage of CD4^+^CD25^+^Foxp3^+^ cells among lymphocytes or a percentage of Foxp3-expressing cells among CD4^+^CD25^+^ cells.

### RNA extraction and quantitative real-time reverse-transcription polymerase chain reaction (RT-qPCR)

Total RNA was isolated from MLNs using the Trizol reagent (Invitrogen, Carlsbad, CA). RNA (1 µg) was reverse-transcribed using random hexamer primers with a complementary (c)DNA synthesis kit (Fermentas, Glen Burnie, MD) according to standard protocols. A real-time RT-PCR was carried out in optical 96-well plates on an ABI 7300 Real-Time PCR System (Applied Biosystems, Foster City, CA). Primers used in this study are listed in Table 2. The expression of each gene was assayed in a total volume of 25 µl containing 1× SYBR green master mix reagent (Applied Biosystems), 100 nM of each primer, and 50 ng of cDNA. Amplification was performed according to the thermocycling protocol recommended by the PCR system, with a final dissociation curve (DC) analysis. No-template controls and a melting curve analysis were used to confirm the specificity of the real-time PCR. All samples were analyzed in triplicate, and multiples of change of messenger (m)RNA were calculated by the equation 2^−ΔΔCt^ (ΔCt indicates the difference of threshold cycles between the test gene and 18 s, and ΔΔCt indicates the difference of ΔCt between the colitis and normal control groups).

**Table 2:** Sequences of oligonucleotide primers used in the PCR amplification.

Gene	Primer sequences (5′ to 3′)
T-bet	F: TGTTCCCAGCCGTTTCTACC
	R:GCTCGGAACTCCGCTTCATA
GATA-3	F: TGACGGAAGAGGTGG ACGTA
	R:GGATACCTCTGCACCGTAGC
RORγt	F: TGCAAGACTCATCGACAAGG
	R: AGGGGATTCAACATCAGTGC
AhR	F: AGGATCGGGGTACCAGTTCA
	R:CTCCAGCGACTGTGTTTTGC
Foxp3	F: AGCGAGTGTCCCTGCTCTCCC
	R: CTTCTGTCTGGAGTGGCTGGGTGT
Muc2	F: ATGCCCACCTCCTCAAAGAC
	R: GTAGTTTCCGTTGGAACAGTGAA
Tff3	F: TTGCTGGGTCCTCTGGGATAG
	R: TACACTGCTCCGATGTGACAG
Hsp72	F: GCTGGCTAGGAGACAGATATGTGGC
	R: AAAGCCCACGTGCAATACACAAAGT
Bcl-xL	F: GGCACTGTGCGTGGAAAGCGTA
	R: CCGCCGTTCTCCTGGATCCA
18 s	F: CGCGGTTCTATTTTGTTGGT
	R: AGTCGGCATCGTTTATGGTC

T-bet, T-box expressed in T cells; ROR-γt, RAR-related orphan receptor gamma t; AhR, aryl hydrocarbon receptor; Foxp3, forhead box p 3.

### Immunoglobulin (Ig) and cytokine concentrations in CLF

Ig, macrophage chemoattractant protein (MCP)-1 and tumor necrosis factor (TNF)-α were determined by enzyme-linked immunosorbent assay (ELISA) kits. Antibodies specific for IgA, IgG, MCP-1 and TNF-α (eBioscience) were coated onto wells of microtiter strips provided. Procedures followed the manufacturer's instructions. Levels of Igs and cytokines were determined by detecting an Ab conjugated to horseradish peroxidase.

### Immunoblotting

Colon tissues were homogenized in lysis buffer (50 mMTris (pH 7.4), 1% sodium dodecylsulfate (SDS), and 10 mM EDTA) containing a protease inhibitor cocktail (Sigma) to prepare whole-tissue lysates. The homogenates were centrifuged at 15,000× g for 15 min, and the supernatants were used for immunoblotting. Protein concentrations of the supernatant were determined using a Bradford Protein Assay Reagent kit (Bio-Rad, Richmond, CA). Twenty micrograms of protein was loaded and separated on 10% SDS–polyacrylamide gel electrophoresis (PAGE) and then transferred to a polyvinylidene difluoride (PVDF) membrane in a wettransfer apparatus. Membranes were blocked with 5% nonfat milk in Tris-buffered saline (TBS) for 30 min and then incubated with antibodies overnight at 4°C. Polyclonal rabbit antibodies against poly (adenosine diphosphate-ribose) polymerase (PARP) were purchased from Cell Signaling Technology (Danvers, MA). Polyclonal rabbit antibodies against nuclear factor (NF)-κB p65 or IκBα were purchased from Santa Cruz Biotechnology (Santa Cruz, CA). A monoclonal mouse antibody against β-actin was purchased from Sigma. After washing 3 times in TBS-T (TBS containing 0.1% Tween-20), membranes were incubated with horseradish peroxidase (HRP)-conjugated species-specific antibodies for 1 h. Membranes were then washed with TBS-T for 30 min, and blots were developed with the high-sensitivity chemiluminescence substrate, Western Lighting Ultra (PerkinElmer Life Sciences, Waltham, MA), and exposed to X-ray films. The relative intensity was measured to quantify the protein level using Image-Pro Plus software (Media Cybernetics, Bethesda, MD). All blots were normalized against actin to adjust for the amount of protein loaded.

### Histopathology

After being embedded in paraffin, specimens of the distal colon were sectioned at 5 µm, mounted on glass slides and stained with hematoxylin and eosin for the histopathology analysis. Digital images at 40× magnification per section were captured with a Zeiss Axiphot light microscope (Carl Zeiss MicroImaging LLC, Thornwood, NY) and a Nikon D1X digital camera (Tokyo, Japan). Five fields per section were examined to determine the morphological lesions and changes in the colon mucosa. The degree of IBD was measured by the modified scoring system of Iba et al. [23]. Inflammatory colitis was scored from 0 to 3 for lesions based on loss of epithelium, length of crypts, and infiltration of leukocytes (Table 3). The total histological score ranged from 0 to 9, which represented the summed scores of loss of epithelium, length of crypts and infiltration of leukocytes, with a higher score indicating more-severe disease.

**Table 3:** Histological score of colitis.

Feature	Score	Description
Loss of epithelium	0	None
	1	0%∼25% loss of epithelium
	2	26%∼50% loss of epithelium
	3	>50% loss of epithelium
Length of crypts	0	None to length of crypts 85% normal thickness
	1	Length of crypts 85%∼70% normal thickness
	2	Length of crypts 70%∼50% normal thickness
	3	Length of crypts <50% normal thickness
Infiltration of leukocytes	0	None
	1	Mild
	2	Moderate
	3	Severe

### Statistical analysis

All data are expressed as the mean ± standard error of the mean (SEM). Differences among groups were analyzed by an analysis of variance (ANOVA) using Tukey's test. A two-way ANOVA using the Bonferroni posttest was used to analyze differences among groups. A *p* value of <0.05 was considered statistically significant.

## Results

### BW changes of the mice

There were no differences in the initial BWs among the 4 groups. Clinical symptoms of exposure to DSS for 5 d were BW loss and the presence of blood in the feces. The BWs did not differ between the C and G groups during the experimental period (data not shown). Weight loss was observed at day (d)11 in the DC group compared to the C group (20.7±1.7 vs. 24.5±0.6 g, *p*<0.05). Also, the BW of the DC group was less than that of the DG group on d11 (20.7±1.7 vs. 22.9±1.4 g, *p*<0.05).

### Colon weight and length, Ig, cytokines and plasma haptoglobin concentrations

There were no differences in colonic weight or length, Ig in lavage fluid, or plasma haptoglobin concentrations between the C and G groups. Mice that received DSS had a significantly shorter colon length, and the DC group had a higher colon weight than the C group. Also, the DC group had significantly higher colonic IgG and plasma haptoglobin levels than those of the control and DG groups. MCP-1 levels were significantly higher in the DC group than the C group. Also, the DC group had higher TNF-α levels than the C and DG group (Table 4).

**Table 4:** Colonic length and weight, and immunoglobulin (Ig) levels in lavage fluid and haptoglobin concentrations in plasma of experimental mice.

	C	G	DC	DG
Colon				
Length (cm)	5.76±0.40	5.53±0.32	4.63±0.69*	4.95±0.49*
Weight (g)	0.23±0.03	0.26±0.04	0.30±0.04*	0.26±0.05
IgA (µg/mg protein)	15.01±0.06	15.02±0.06	17.18±0.54	16.85±0.52
IgG (µg/mg protein)	20.53±2.77	17.00±4.38	105.53±4.17* #	41.23±6.44
MCP-1 (pg/ml)	60.30±1.48	63.62±1.17	102.6±2.70*	74.06±0.68
TNF-α (pg/ml)	43.52±0.71	41.85±0.16	61.70±1.01* #	48.38±1.44
Plasma				
Haptoglobin (µg/ml)	14.43±0.14	15.31±0.42	121.21±8.50* #	40.87±1.8

Data are expressed as the mean ± SEM. Differences among groups were analyzed by ANOVA using Tukey's test.

Significantly differs from the C group.

^#^ Significantly differs from the DG group (*p*<0.05).

### Percentage of blood T cell subpopulations

**Figure 1:**
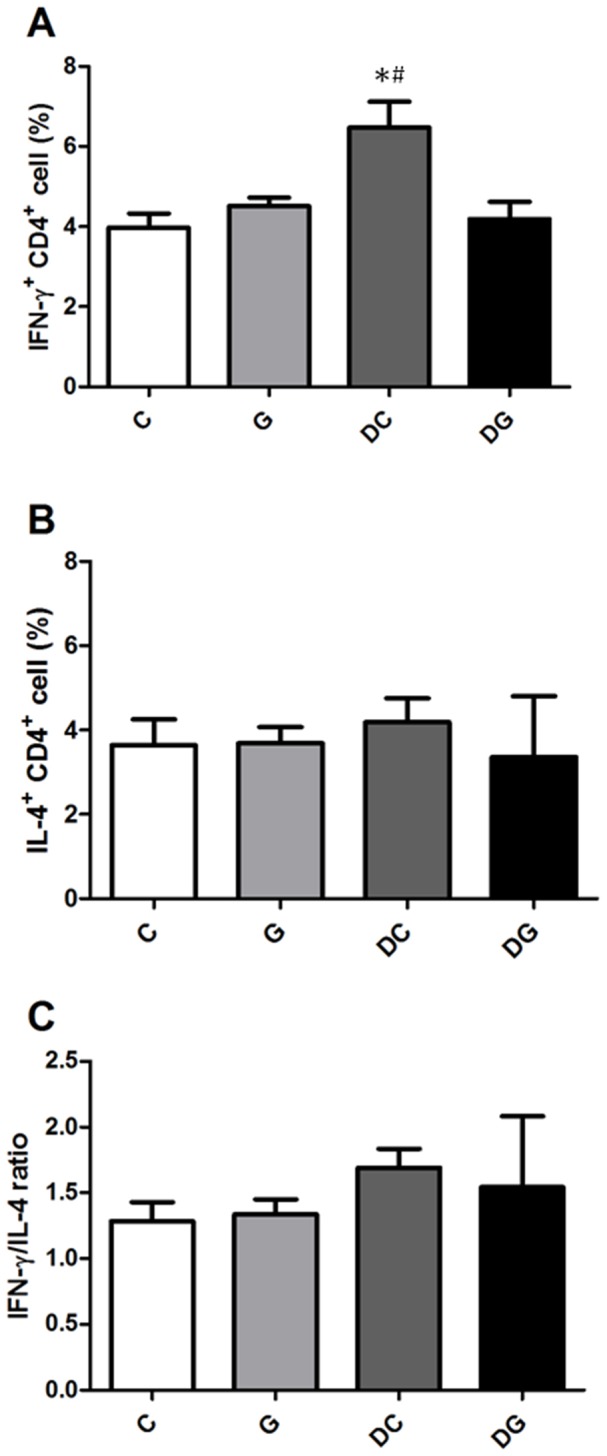
Percentage of Th1/Th2 cell subpopulations in the blood. Lymphocytes were gated according to their size and granularity using light scatter detectors (FSC/SSC). CD4-positive lymphocytes were considered Th cells and were gated to determine the expression of intracellular cytokines. A, B: Percentages of interferon (IFN)-γ- and interleukin (IL)-4-expressing CD4^+^ lymphocytes. C: IFN-γ/IL-4 ratio. Data are presented as the mean ± SEM. Differences among groups were analyzed by ANOVA using the Tukey's test. *Significantly differs from the C group. ^#^Significantly differs from the DG group (*p*<0.05).

**Figure 2:**
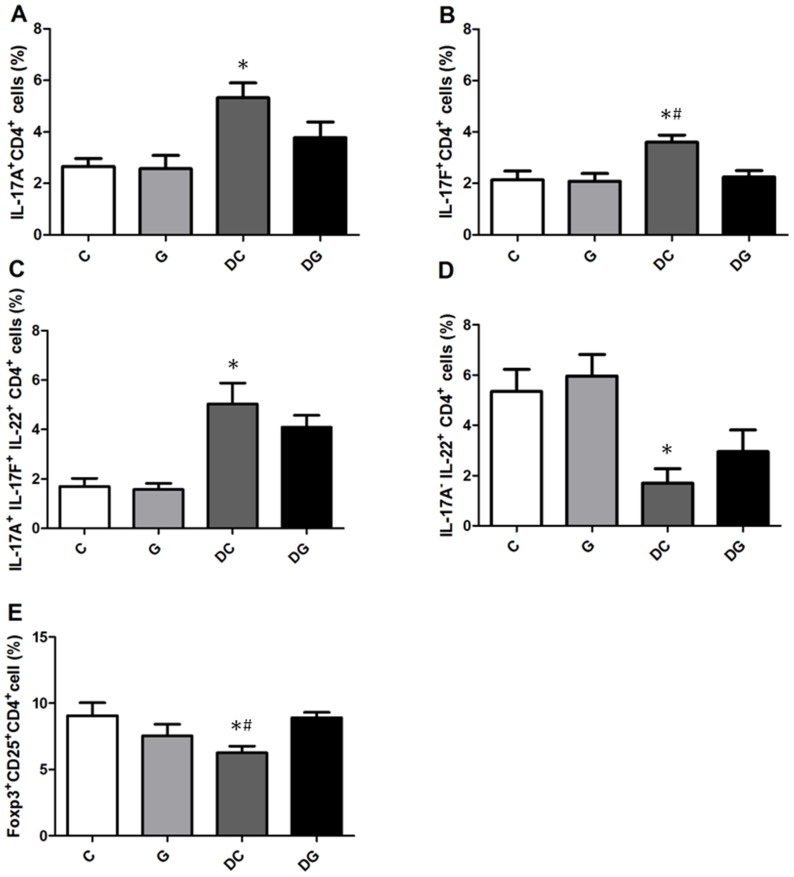
Percentages of Th17/Th22-associated cytokine-producing cell and Treg cell populations in the blood. CD4-positive lymphocytes were gated to analyze intracellular cytokine expressions by flow cytometry. A: Expression of intracellular interleukin (IL)-17A. B: Expression of intracellular IL-17F. C: Triple expression of intracellular IL-17A, IL-17F, and IL-22. D: IL-22^+^ and IL-17A^−^Th cells. E: Percentage of Treg cells. Data are presented as the mean ± SEM. Differences among groups were analyzed by an ANOVA using Tukey's test. *Significantly differs from the C group. ^#^Significantly differs from the DG group (*p*<0.05).

There were no differences in percentages of Th cell subpopulations between the C and G group. Percentages of IFN-γ-expressing CD4^+^ cells were higher in the DC group than in the C and DG groups. No differences in IL-4-expressing CD4^+^ cells or the IFN-γ/IL-4 ratio were detected among the 4 groups (Figure 1). The DC group had significantly higher percentages of IL-17A, IL-17F, and IL-17A^+^IL-17F^+^IL-22^+^-expressing CD4^+^ cells while lower IL-17A^−^IL-22^+^ Th22 cells than those in the C group. Also, Foxp3^+^CD4^+^CD25^+^ Treg cells were lower in the DC group than the C and DG groups. The DG group had lower IL-17F-expressing CD4^+^ cells and higher Foxp3^+^CD4^+^CD25^+^ Treg cells than the DC group but showed no differences from the C group (Figure 2).

### Gene expressions in MLNs and colon tissues

**Figure 3:**
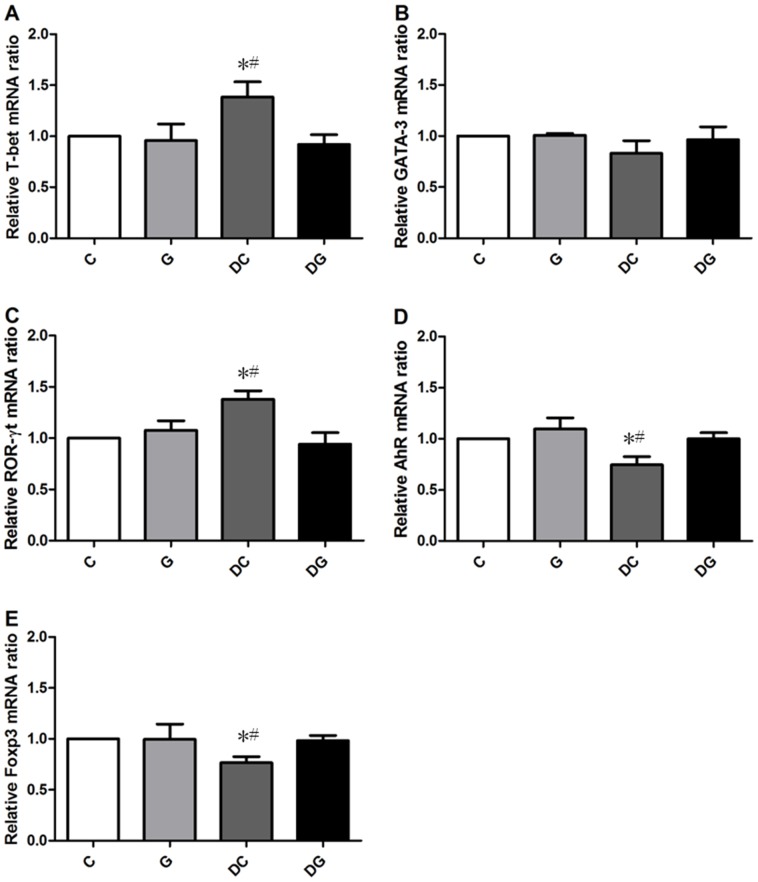
Transcription factor gene expressions of A) T-bet, B) GATA-3, C) ROR-γt, D) the aryl hydrocarbon receptor (AhR), and E) forhead box p3 (Foxp3) in mesenteric lymph nodes (MLNs) were analyzed by a real-time PCR. Quantitation of mRNA changes was calculated by the comparative CT (2^−ΔΔCt^) method, and the mRNA expression of C mice was used as a calibrator. Data are shown as the mean ± SEM. *Significantly differs from the C group. ^#^ Significantly differs from the DG group (*p*<0.05).

**Figure 4:**
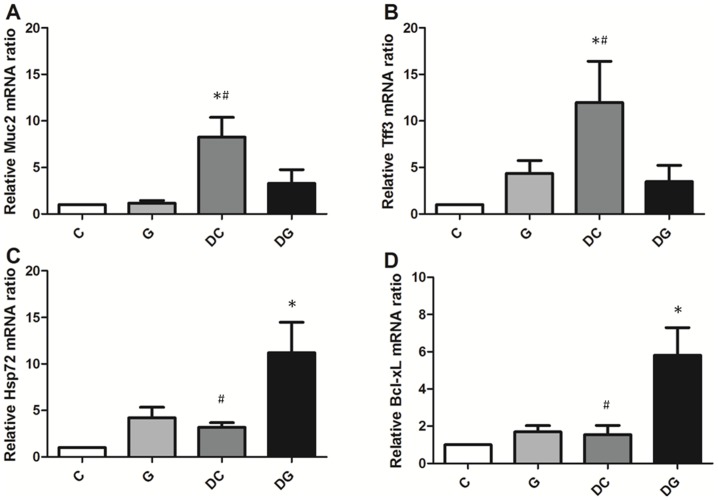
mRNA expressions of mucosal recovery-related and antiapoptotic genes in colon tissues. mRNA levels were analyzed by a real-time PCR. A: Expression of Muc2 mRNA. B: Expression of Tff3 mRNA. C: Expression of Hsp72 mRNA. D: Expression of Bcl-xL mRNA. Quantitation of mRNA changes was calculated by the comparative CT (2^−ΔΔCt^) method, and mRNA expression of C mice was used as a calibrator. Data are shown as the mean ± SEM. *Significantly differs from the C group. ^#^Significantly differs from the DG group (P<0.05).

mRNA levels of T-box expressed in T cells (T-bet) and RAR-related orphan receptor gamma t (ROR-γt) were higher whereas the aryl hydrocarbon receptor (AhR) and Foxp3 gene expressions in MLN were lower in the DC group than in the C and DG group (Figure 3). Mucin 2 (Muc2) and trefoil factor 3 (Tff3) mRNA levels in the colon tissues were significantly higher in the DC group than the other 3 groups. The DG group had the highest gene expressions of Hsp72 and antiapoptotic Bcl-xL in colon tissues among the 4 groups (Figure 4).

### Protein levels of PARP and NF-κB/IκBα in colon tissues

**Figure 5:**
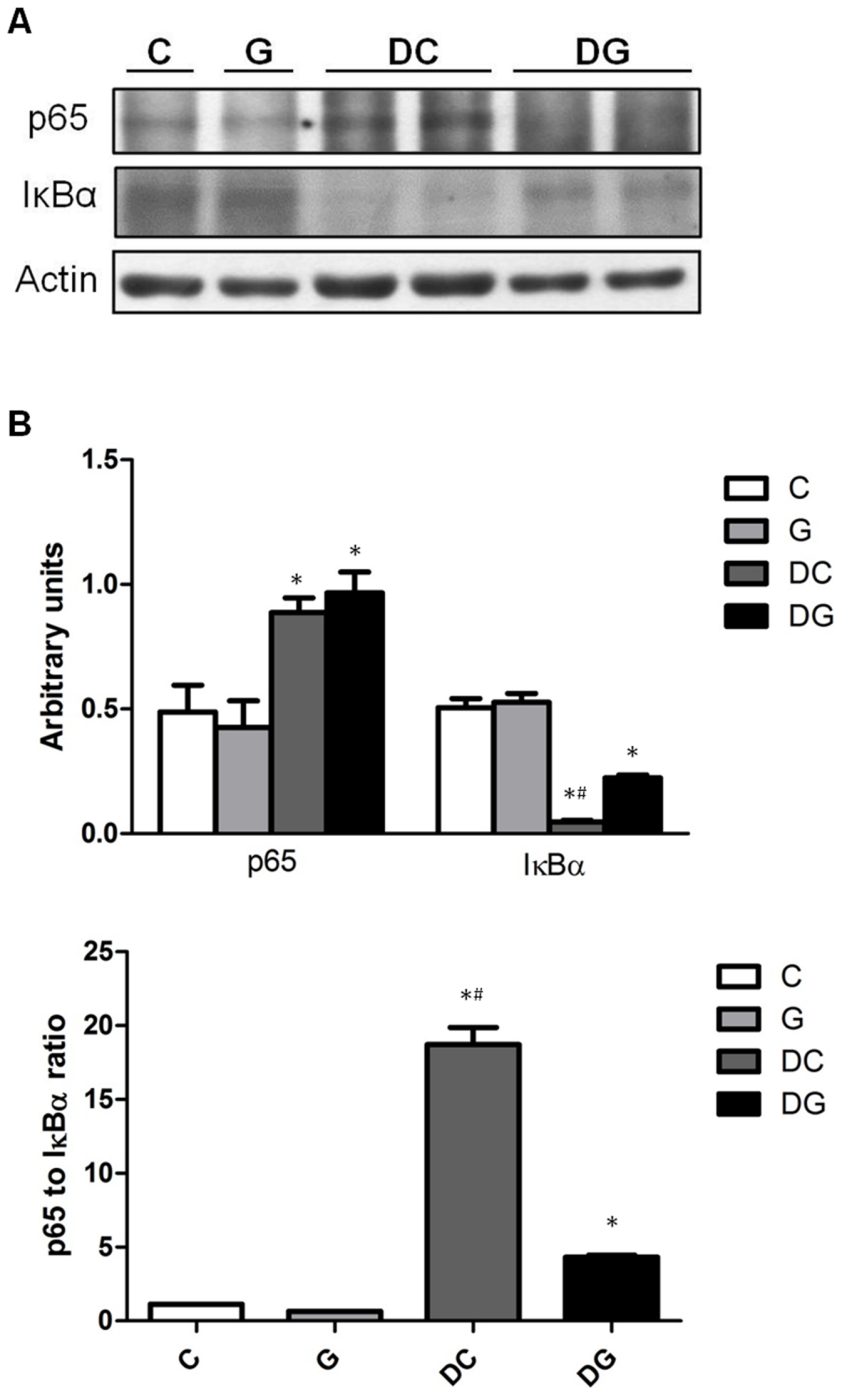
Protein levels of nuclear factor (NF)-κB p65 and inhibitory factor κBα (IκBα) in colon tissues. a Protein expressions of NF-κB p65 and IκBα. Whole-tissue lysates were analyzed by immunoblotting, and b-actin was used as a loading control. b Densitometric analysis of the blot corrected by the protein loading control. c The ratio of NF-κB p65 to IκBα. Results of the densitometric analysis are shown as the mean ± SEM. *Significantly differs from the C group. ^#^Significantly differs from the DG group (P<0.05).

**Figure 6:**
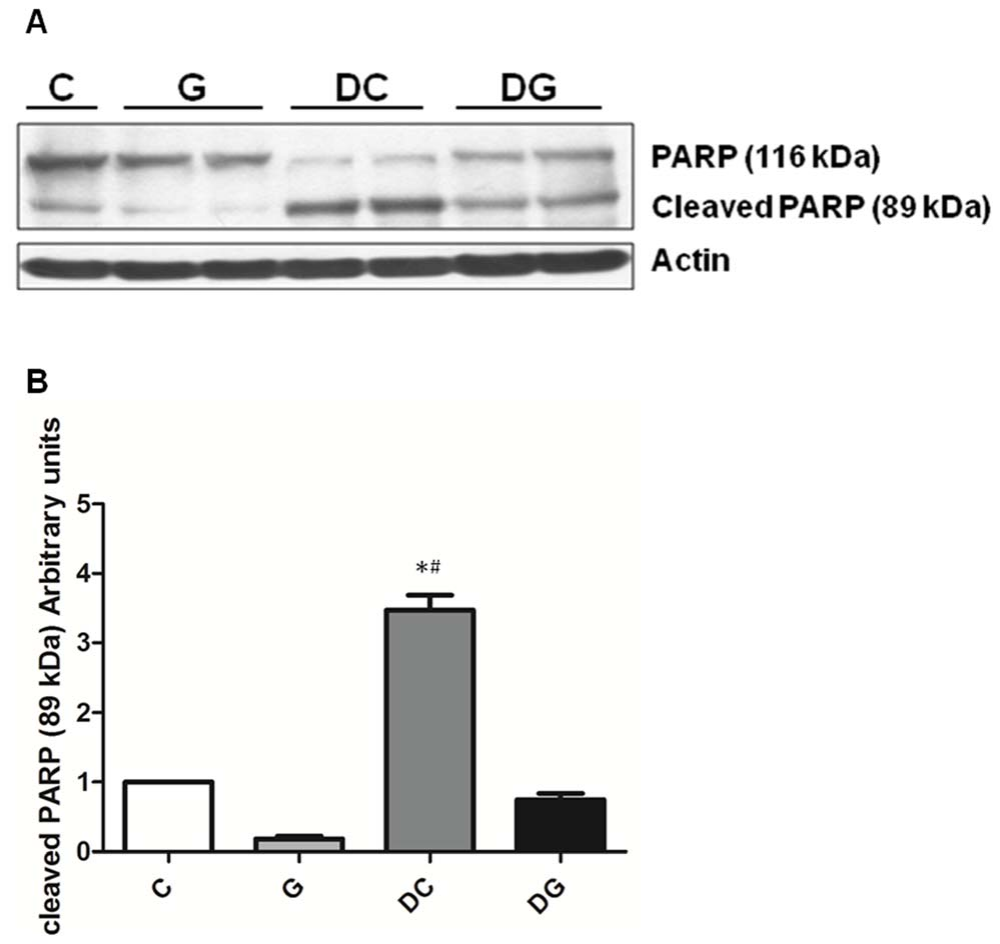
Protein levels of PARP and cleaved PARP in colon tissues. A: protein expressions of PARP and cleaved PARP. Whole-tissue lysates were analyzed by immunoblotting, and β-actin was used as a loading control. B: densitometric analysis of the blot corrected by the protein loading control. Results of the densitometric analysis are shown as the mean ± SEM. *Significantly differs from the C group. ^#^Significantly differs from the DG group (P<0.05).

Compared to the NC group, the DS and DG groups had significantly higher NF-κB p65 whereas lower IκBα expressions. The NF-κB/IκBα ratio was lower in the DG group than the DC group (Figure 5). DC group showed significantly higher protein levels of cleaved PARP than the C and DG group (Figure 6).

### Histopathological aspects of the colon

**Figure 7:**
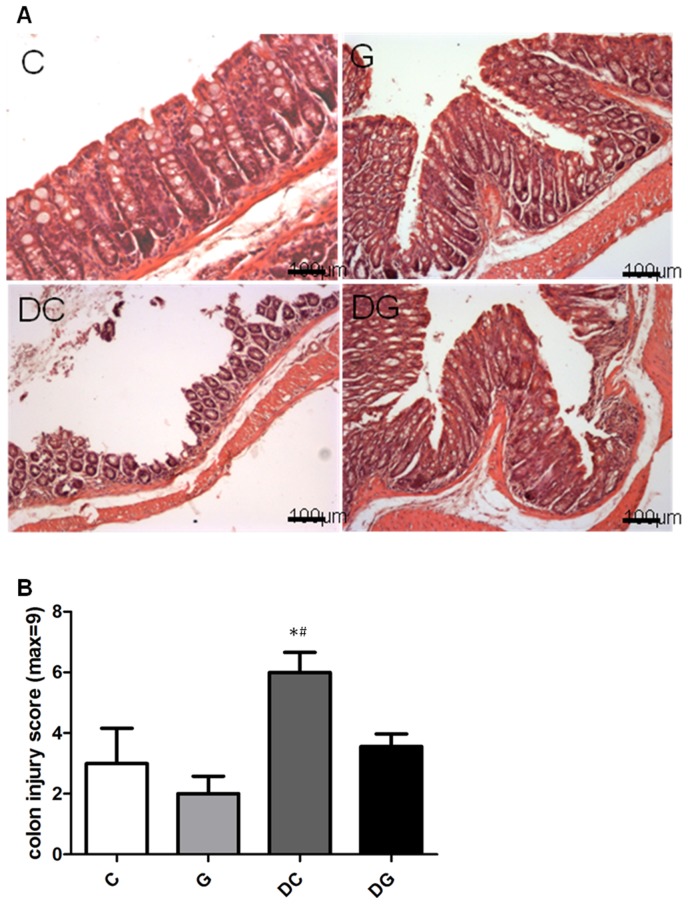
Histopathology of colon tissues. A: Hematoxylin and eosin staining of colon tissues from mice in the C, G, DC, and DG groups. B: Histological scores of colitis. Data are presented as the mean ± SEM., which were determined as described in “Materials and Methods.” Differences among groups were analyzed by a one-way ANOVA using Tukey's test. *Significantly differs from the C group. ^#^Significantly differs from the DG group (*p*<0.05).

Colon section from the C and G groups showed intact epithelium, a well-defined gland, and no leukocyte infiltration in the mucosa. In contrast, the DC group showed mucosal ulceration, infiltration of leukocytes, crypt distortion, and hyperplastic epithelium, and had a significantly higher colon injury score than the C and DG groups (Figure 7).

## Discussion

In this study, dietary Gln was administered for 5 d before colitis was induced, and was sustained through the period of DSS-induced colitis. Because IBD is a disease that flares up with remissions and relapses, this model mimics the preventive oral Gln administration to the acute phase of IBD patients in order to resolve the inflammation of colitis. We did not include an amino acid mixture without Gln group in this study, because previous study had shown that Gln, but not isonitrogenous non-amino acid mixture, promotes T lymphocyte response [24]. A study conducted by our laboratory previously compared intraperitoneal injection of Gln dipeptide and identical amount of amino acid solution in a mouse colitis model. Our results also showed that compared with the mixed amino acid solution group, Gln administration reduced inflammatory mediator production and leukocyte infiltration into tissues [21]. We analyzed the distribution of blood T cell populations including Th1, Th2, Th17, Th22, and Treg cells, because the balance between Th and Treg cells and the cytokine profiles derived from these T cells play major roles in the inflammation of IBD [12], [25], [26]. T-bet, GATA-3, ROR-γt, AhR, and Foxp3 are transcription factors that respectively participate in the differentiation of Th1, Th2, Th17, Th22, and Treg cells. Since the colon is the main site of injury in IBD, gene expressions of transcription factors in MLNs were also analyzed to evaluate the polarization of Th subsets and Treg cells. The findings of this study showed that dietary Gln supplementation before and during colitis modulate the homeostasis of Th and Treg cells that may mitigate colonic inflammatory reactions. Besides, the partial elemental component provided by Gln diet may also have favorable effect on treating IBD.

Cytokines are key players in innate and adaptive immune responses in mucosal inflammation. A previous study indicated that different models of IBD produced specific cytokine profiles in the initiation and perpetuation of disease pathogenesis [27]. Those investigators suggested that cytokine profiling can be used as diagnostic and prognostic tools in IBD. Our results showed that percentages of blood IFN-γ, IL-17A, and IL-17F together with the mRNA expressions of T-bet and ROR-γt in MLNs were upregulated in the DC group. This result is consistent with a previous report which also showed that DSS-induced acute colitis was characterized by polarization toward the Th1-Th17 panel [27]. Th22 is a newly identified subset of Th cells. Th22 appears to be regulated by the AhR transcription factor and plays a critical role in mucosal healing through epithelial proliferation and increased mucus production. Activation of the AhR ameliorates the inflammation and disease activity of colitis mice [28]–[30]. Our results showed that DSS administration reduced the population of blood Th22 cells and mRNA levels of the AhR in MLNs indicating that Th22 expression was suppressed in mice with acute colitis. These findings were consistent with previous studies that Th22 and AhR expressions are downregulated in actively inflamed colitis tissue of IBD patients and experimental colitis models [9], [29].

Treg cells express the CD4 and CD25 surface markers. Foxp3 is a key transcription factor of Treg cell development and function, and is the best marker for defining murine Treg cells [10], [31]. Treg cells were shown to suppress a number of additional T cell-mediated responses in vitro and in vivo and are effective in the prevention and downregulation of IBD in animal models [32]–[34]. Results of this study found that percentages of blood Treg cells and mRNA levels of Foxp3 in MLNs significantly decreased in the DC group. Our findings are consistent with previous reports which also found that Treg cells decreased in the blood and inflamed colon tissue of IBD patients [13], [35]–[37].

Haptoglobin is an acute-phase protein, which is used as a marker of systemic inflammation to monitor disease activity in experimental colitis in rodent models [38]. IgA is the main Ig produced by the intestinal mucosa. In this study, IgA levels did not differ among the control and DSS colitis groups. This result is similar with a report by Macpherson et al. [39], who also found no difference in colonic IgA concentrations between IBD patients and controls. In contrast to IgA, IgG is considered a specific index for grading disease activity in patients with IBD [40]. We also found dramatically higher levels in the DSS colitis group. MCP-1 is a chemotactic and activating factor for mononuclear phagocytes. MCP-1 and TNF-α are important mediators involved in the inflammatory and immune responses. NF-κB is a widely expressed inducible transcription factor that is an important regulator of genes involved in inflammatory response. NF-κB DNA binding is inhibited by IκBα. The higher NF-κB/IκBα ratio indicates higher NF-κB activation. The significantly elevated plasma haptoglobin and intraluminal mediators accompanied with higher NF-κB/IκBα ratio indicated that experimental colitis was active.

Previous study demonstrated that DSS results in epithelial damage by inhibiting proliferation and inducing apoptosis [41]. There is a layer of mucus formed by mucins lining the gastrointestinal tract to act as a lubricant and provide protection of mucosal barrier. Muc2 is one of the predominant mucins detected in the colorectum. Trefoils are a group of small peptides that have important roles in epithelial protection and mucosal healing [42]. Trefoil factor family (Tff)3 is one of the Tff peptides found throughout the small and large intestines. Previous studies found that Muc2 and Tff3 genes were up-regulated during colitis induction, and suggested that their mRNA levels could be used as early markers of inflammation [43]. The higher Muc2 and Tff3 mRNA expressions in the DC group may indicate more severe mucosal damage that required to be repaired. Apoptosis is a form of cell death, which is controlled by intracellular proteases of the caspase family. Caspases cleave various cellular substrates including PARP and thereby induce the typical alterations of apoptotic cells [44]. The result of this study showed that the protein levels of cleaved PARP in colon tissue were elevated in the DC group suggested DSS-induced apoptosis is initiated.

In this study, we found that dietary Gln supplementation resulted in several beneficial effects that were not observed in colitis mice without Gln administration. First, the DSS-induced Th1–Th17 response and decreased Th22 and Treg cell expressions were inhibited by Gln administration. These findings were partly similar to a previous study which used an intraperitoneal injection of Gln in mice with DSS-induced colitis [21]. Second, transcription factor gene expressions corresponding to Th and Treg cells in MLNs were consistent with the T cell response in the blood indicating that Gln has a regulatory role in systemic and local T cell polarization in acute colitis. Third, dietary Gln supplementation lowered the plasma haptoglobin and intraluminal inflammatory mediators. The lower NF-κB/IκBα ratio further supports the role of Gln in reducing colitic inflammation. Fourth, Gln prevented the apoptosis of colon cells possibly by reducing caspase activities and enhancing colonic antiapoptotic Bcl-xL gene expression. The higher heat shock protein (Hsp) expression may also play roles in inhibiting apoptosis. Hsp is vital to cellular and tissue protection after stress or injury. Previous study indicated that Gln protected gut mucosa from injury by inducing Hsp72 expression [45], [46]. The chaperone property of Hsp72 is related to the cytoprotective effects and the ability to inhibit apoptosis [47], [48]. The lower colonic Muc2 and Tff3 mRNA expressions observed in the DSS-Gln group were consistent with the histological findings that damage to the colonic mucosa was less severe in the colitis group with Gln supplementation.

Gln is the primary fuel source for immune cells. Also, it is a precursor for the endogenous antioxidant, glutathione. Previous studies demonstrated that Gln enhanced IL-2 production, promoted T cell proliferation, and decreased T cell apoptosis possibly through certain redox-based mechanisms [49], [50]. In addition, IL-2 was found to maintain Foxp3 Treg cells and inhibit the polarization of Th17 cells [51], [52]. However, elucidating the mechanisms responsible for the effects of Gln on regulating T cell polarization and the association between CD4+ T cell homeostasis and inflammatory response requires further investigation.

In summary, this study showed for the first time that in a DSS-induced acute colitis model, dietary Gln supplementation suppressed Th1/Th17-associated cytokine expressions while enhancing Th22 and Treg cell expressions. Also, systemic and intraluminal inflammatory proteins and the apoptosis of colonic cells were reduced. These results suggest that Gln treatment modulates the homeostasis between Treg and Th cells, and ameliorates the disease severity of acute DSS-induced colitis.
